# The Natural Compound Hydrophobic Usnic Acid and Hydrophilic Potassium Usnate Derivative: Applications and Comparisons

**DOI:** 10.3390/molecules26195995

**Published:** 2021-10-02

**Authors:** Hallysson Douglas Andrade de Araújo, Hianna Arely Milca Fagundes Silva, José Guedes da Silva Júnior, Mônica Camelo Pessoa de Azevedo Albuquerque, Luana Cassandra Breitenbach Barroso Coelho, André de Lima Aires

**Affiliations:** 1 Centro de Biociências-Departamento de Bioquímica, Universidade Federal de Pernambuco (UFPE), Avenida Prof. Moraes Rego, 1235, Cidade Universitária, Recife 50670-501, PE, Brazil; hiannaamfs@gmail.com (H.A.M.F.S.); zeguedescrizant@gmail.com (J.G.d.S.J.); 2 Laboratório de Imunopatologia Keizo Asami (LIKA), Universidade Federal de Pernambuco, Avenida Prof. Moraes Rego, 1235 Cidade Universitária, Recife 50670-501, PE, Brazil; jcmonica@globo.com; 3 Centro de Ciências Médicas–Departamento de Medicina Tropical, Universidade Federal de Pernambuco, Avenida Prof. Moraes Rego, 1235 Cidade Universitária, Recife 50670-501, PE, Brazil

**Keywords:** lichen, secondary metabolite, usnic acid, acid-base reaction

## Abstract

Usnic acid is the best-studied lichen metabolite, presenting several biological activities, such as antibacterial, immunostimulating, antiviral, antifungal, anti-inflammatory, and antiparasitic agents; despite these relevant properties, it is a hydrophobic and toxic molecule. In this context, scientific research has driven the development of innovative alternatives, considering usnic acid as a source of raw material in obtaining new molecules, allowing structural modifications (syntheses) from it. The purpose is to optimize biological activities and toxicity, with less concentration and/or response time. This work presents a literature review with an analogy of the hydrophobic molecule of usnic acid with its hydrophilic derivative of potassium usnate, emphasizing the elucidation and structural characteristics, biological activities, and toxicological aspects of both molecules, and the advantages of using the promising derivative hydrophilic in different in vitro and in vivo assays when compared to usnic acid.

## 1. Introduction

Usnic acid (2,6-diacetyl-7,9-dihydroxy-8,9b-dimethyl-1,3(2*H*,9b*H*)-dibenzo-furandione); C_18_H_16_O_7_, MW of 344.32 is a molecule metabolized by several species of lichens (symbiotic organisms composed of a fungus (mycobiont) in association with one or more photosynthetic partners (photobiont) which may be green algae, cyanobacteria or both), of the genera *Cladonia*, *Usnea*, *Lecanora*, *Ramalina*, *Parmelia*, and *Evernia*, widely distributed, present in the arctic, tropical and subtropical countries and the Antarctic [1,2,3,4,5]. Usnic acid is found naturally in two enantiomeric forms, such as (−) levogyrous and (+) dextrogyrous, due to the angular projection of the methyl group located in position 9b [6,7] being still characterized as a yellow pigmented substance; its forms can be varied, that is, depending directly on the solvent used in its recrystallization [8,9].

Although usnic acid is a molecule with an initial report available in the literature in the 1950s, whose purpose was the search for new antibiotics [10], even nowadays this molecule is used in pharmaceutical industry applications [11,12,13], with a breakthrough and hundreds of scientific papers to demonstrate the efficacy of the various biological activities and toxicity attributed to usnic acid [4,9]. Antibacterial action [14], gastroprotective effect [15], immunostimulatory [16], antiviral [17], antifungal [18], anti-inflammatory [19], antiparasitic [20] and antitumor activity [21]. Despite the relevant biological effects, usnic acid is a hydrophobic molecule, thus limiting its use in clinical applications [22,23].

Scientific research has driven the development of innovative alternatives, considering usnic acid as a source in obtaining new molecules, allowing structural modifications (syntheses) from usnic acid, whose purpose is to potentiate biological activity, leading to an optimization of therapeutic activity [24,25]. Our research group has expertise with relevant scientific productions with usnic acid [26,27,28,29,30,31,32,33] and the production/synthesis of its derivative, potassium usnate [34,35,36,37,38,39,40]. This article aimed to describe the research found in the literature, the following databases were used: MEDLINE, Cochrane Central Register of Controlled Trials (CENTRAL/CCTR), Multidisciplinary Digital Publishing Institute (MDPI), Scientific Electronic Library Online (SciELO), Health of Latin America and the Caribbean Scientific Literature (LILACS), Google Scholar, and reference lists of relevant articles making an analogy of the molecule of usnic acid with potassium usnate, emphasizing the elucidation and structural characteristics, biological activities, bioavailability and toxicological aspects of both molecules and the advantages of using the promising hydrophilic derivative in different in vitro and in vivo assays when compared to usnic acid.

## 2. Chemical Properties of Usnic Acid and Potassium Usnate

**Figure 1:**
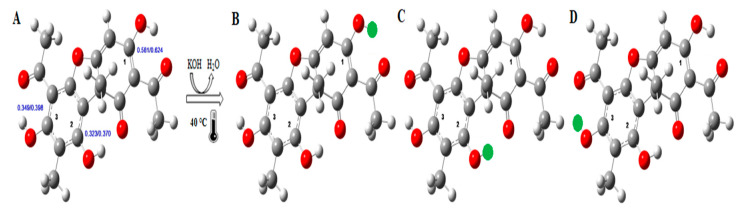
Structure of the usnic acid (**A**), synthesis reaction forms of the structures of the usnic acid anions (**B**–**D**), after a single deprotonation in carbons 1, 2 and 3, respectively. Color of the spheres corresponding to the chemical elements of the structures of usnic acid and potassium usnate: dark gray carbon, red carbon, light gray hydrogen and green potassium.

The usnic acid molecule is insoluble in water and glycerol, partially soluble in ethanol and very soluble in ether, acetone, benzene, and chloroform [22,23]. Its hydrophobic character from the presence of four functional ketone groups and the furan ring that joins the two aromatic rings of the molecular structure [2]. Studies by Buemi and Zuccarello [41], Galasso et al. [42] and Araújo et al. [38], who evaluated the molecular conformations, hydrogen bonding forces and electronic structure of the usnic acid using the semi-empirical method AM1, and the electronic part of the equilibrium geometries obtained according to the ab initio DFT method (Theory of the functional density), identified the most stable tautomer of usnic acid and, from the charges of electrostatic potential, observed that carbon 1 is the most electrophilic compared to carbons 2 or 3, both in gas and in aqueous solution, presenting the values load 0.581/0.624, 0.323/0.370 and 0.349/0.398 for carbons 1, 2 and 3, respectively (Figure 1A).

Potassium usnate is characterized by the presence of the potassium element (K^+^) in the structure of the usnic acid molecule from an acid-base reaction, making it a water-soluble derivative. Huneck and Yoshimura [8] described that the first deprotonation for K^+^ entry occurs in the OH group of C1 carbon, an observation confirmed by Guo et al. [43] when mapping the pKa of the OH groups of the usnic acid molecule, and considered the OH group of C1 more prone to the synthesis reaction (Figure 1B). Araújo et al. [38] compared the sum of the thermal correction term (obtained by semi-empirical method AM1 in the optimization of the molecule geometry) with the electronic energy (DFT/B3LYP) between the usnic acid resulting from the deprotonation of the oxygen linked to the carbons 1 (Figure 1B), 2 (Figure 1C) or 3 (Figure 1D) (leading to anions A1, A2 and A3, respectively) and the molecular masses correspond to 382.20 MW. Both in ideal gas and an aqueous solution, the anion created by the deprotonation of non-phenolic OH (C1) is the most stable (Figure 1B). The following stability orders, with the energies related to such an anion, were observed (in water): (a) A1 > A2 (6.76 kcal·mol ^−1^) > A3 (18.23 kcal·mol ^−1^) in the ideal gas and (b) A1 > A2 (7.81 kcal·mol ^−1^) > A3 (10.94 kcal·mol ^−1^).

## 3. Molluscicidal and Antiparasitic Activities

Freshwater mollusk of the species *Biomphalaria glabrata* (Say, 1818) is considered in South America, mainly in Brazil, the main intermediate host of the *Schistosoma mansoni*. *B*. *glabrata* has great susceptibility to infection, efficacy in the transmission of schistosomiasis, releasing thousands of cercariae daily in the aquatic environment, wide geographical distribution, long life span, and short embryonic cycle [44,45,46]. Molluscicidal activity using the usnic acid from *Cladonia substellata* and solubilized in DMSO (0.5%) was reported by Araújo et al. [26,27] on the different embryonic stages and adult snails of *B. glabrata*. The toxic and teratogenic effects of usnic acid in the stages of blastula, gastrula, trocophore and veliger after 24 h of exposure corresponded to an LC_50_ and LC_90_ of 1.38 and 1.62, 3.47 and 4.45, 5.11 and 5.36 and, finally, 2.93 and 4.49 μg/mL, respectively. While, for adult *B. glabrata*, the LC_50_ and LC_90_ after 24 h of exposure to the usnic acid corresponded to 2.12 and 3.45 μg/mL, respectively. Regarding the evaluation of the activity of the usnic acid (DMSO 0.5%) isolated from *C. substellata* on cercariae of *S. mansoni* exposed for 2 h, Carvalho et al. [47] observed that the concentration of 10 μg/mL caused 100% mortality in 90 min of exposure, while the concentration of 7.5 μg/mL showed mortality greater than 50% in the first 30 min; at a concentration of 1 μg/mL, cercariae remained viable with a low percentage of mortality after 2 h (Table 1).

Molluscicide and cercariae assays were also evaluated against potassium usnate under the same experimental protocol for the embryonic stages and adult mollusks of *B. glabrata* as for the infectious agent of schistosomiasis mansoni were also reported. Araújo et al. [35,36] demonstrated that the embryonic stages exposed to potassium usnate showed greater susceptibility, when compared to usnic acid, except for the blastula phase only, presenting the following LC_50_ and LC_90_ for the embryonic stages, blastula 4.97 and 5.41 μg/mL, gastrula 3.11 and 3.58 μg/mL, trocophore 3.55 and 4.44 μg/mL and finally, speed 2.67 and 3.89 μg/mL. Whereas, for adult mollusks, only the LC_50_ was determined at 9.2 μg/mL, reaching lethality for all adult mollusks of *B. glabrata* at 1 μg/mL [34]. The restricted activity of potassium usnate at a concentration of 5 μg/mL caused 100% lethality in 90 min of exposure, while in the concentration of 2.5 μg/mL it presented a lethality equal to or greater than 90% after 2 h of exposure. The authors also reported that the cercariae that were alive after 2 h exposure in concentrations 1.5, 1 and 0.5 μg/mL had altered motility with rotation on the axis itself, slow rhythm and contortions, characteristics that are unfavorable to the infectious capacity of the cercariae [35,36] (Table 1).

*S. mansoni* etiologic agent of schistosomiasis mansoni is the most widespread species within its genus in the world, present in 52 countries and territories in tropical and subtropical regions, especially in the Americas, where the presence of this single species is reported [48,49]. Salloum et al. [50] evaluated the in vitro activity of isolated usnic acid from *Usnea steineri* on *S. mansoni* couples and reported 100% mortality from worms only at the concentration of 200 μM and in the largest exposure interval 120 h and a complete reduction in fertility/egg production for the same observation interval (Table 1). In addition, through scanning electron microscopy (SEM), discrete integumentary changes in the parasites have been reported, highlighting only peeling and the presence of bubbles.

Potassium usnate showed a very promising in vitro schistosomicidal effect, with lethality for couples of *S. mansoni* adult worms at concentrations 100, 50, 25 and 12.5 μM, in the observation intervals at 24, 48, 96 and 120 h, respectively. The authors noted that 50% less of potassium usnate (100 μM) and a 5-fold shorter interval (24 h) were needed to achieve lethality of all parasites when compared to usnic acid (Table 1). Regarding fertility, females of *S. mansoni* exposed to potassium usnate showed zero capacity, since no eggs were observed in any concentration in the different intervals. Concerning the tegumentary changes in the worm couples, areas with swelling, loss of spines, presence of blisters, dorsoventral contraction, erosion, exposure of the subtegumentary tissue, disintegration of the tubers and integument were evidenced [37,39].

Leishmaniasis is a parasitic infection caused by protozoa of the genus *Leishmania* spp. representing a serious public health problem in many countries of the world, affecting socially and economically vulnerable populations [51]. In the study of Luz [52], a comparative analysis of the in vitro activities of the single usnic acid from *C. substellata* and potassium usnate against the promastigotes of *Leishmania* (Leishmania) *infantum chagasi* was carried out, describing the morphological changes of the parasites through SEM, in addition, to assess the cytotoxicity on macrophages (RAW 264.7) for both drugs after exposure. The IC_50_ of the usnic acid on the promastigote forms of the parasite corresponded to 18.30 μM. SEM revealed swelling of the parasites, loss of cell polarity and an increase in the number of cytoplasmic vacuoles. Regarding toxicity to macrophages, the CC_50_ concentration was 3.75 μM. When compared with potassium usnate, it showed leishmanicidal activity with an IC_50_ of 2.99 μM and the SEM images revealed changes in nuclear chromatin, intense cytoplasmic vacuolization, extensive mitochondrial swelling, lipid bodies and electron-dense vesicles throughout the cytoplasm of the parasites and finally, the toxicity on the macrophages corresponded to CC_50_ = 26.31 μM (seven times less) (Table 1).

## 4. Antinociceptive Activity and Cytotoxicity on Peripheral Blood Cells

Pain is an unpleasant sensory and emotional experience associated with actual or potential tissue damage, and it is described in terms of such damage directly associated with different functions of the neurological signal with nociceptive perception due to sensations stimulated by subjective and objective factors of the system [53,54]. In this sense, Okuyama et al. [55] evaluated the usnic acid isolated from *Usnea diffracta* as an analgesic component in a murine pain model by the method of abdominal contortions induced by the intraperitoneal administration of acetic acid at concentrations of 30 and 100 mg/kg. The authors concluded that acetic acid-induced pain to peripheral nociceptors was significantly reduced only at the concentration of 100 mg/kg with a 60% percentage of antinociception when compared to the control group (Table 2). In terms of cytotoxicity on human peripheral blood lymphocytes Prokopiev et al. [56] using the MTT method, observed a substantial cytotoxic action on different concentrations of usnic acid; the percentage of lymphocyte viability for concentrations 0.08, 0.15 and 0.30 mM were approximately 40, 30 and 15% viable lymphocytes.

Araújo et al. [38] evaluated potassium usnate and its antinociceptive activity according to the method previously described [55]. The treatments were carried out in concentrations of 10 and 20 mg/kg of potassium usnate and a very significant inhibition on pain was observed with percentages of 68% and 78%, respectively. The authors attributed the results to the presence of the radical K^+^ present in the structure of the potassium usnate considering that this is the only element that differentiates it from the usnic acid and confers hydrophilic characteristics in the molecule (Table 2). The evaluation of the cytotoxicity of potassium usnate by the MTT method on peripheral blood mononuclear cells (PBMCs) that include lymphocytes and monocytes was reported by Araújo et al. [37]. The authors only reported that the minimum inhibitory concentration to render 50% of PBMCs unfeasible was above the investigated concentration (IC_50_ > 200 μM).

## 5. Antitumor Activity and Bioavailability

Cancer is a term used to describe more than 100 different diseases that have in common the fact that they are the result of an uncontrollable growth of cells, which can invade neighboring tissues [57]; colorectal cancer is among the most common types [58]. In this approach, Yang et al. [59] evaluated the anticancer activity of usnic acid (Sigma, St. Louis, MO, USA) and potassium usnate in an in vitro and in vivo experimental model of colorectal cancer for the oral bioavailability of both molecules.

In in vitro assays, usnic acid showed cytotoxic activity (assessed by the MTT method) on different human colorectal cancer cell lines (DLD1, SW480, HT29, SW620, COLO320, Caco2 and HCT116,) and murine carcinoma (CT26) in concentrations 12.5–100 µM. It was also observed that the last three cancer cell lines (human and murine) chosen had a lower number of invaded cells in the groups treated with the usnic acid, in the concentration of 5 μM, when compared with the control groups (Table 1).

In the in vivo assay, CT26 cells expressing luciferase were inoculated by splenic injection to form multiple tumors in the liver of syngeneic BALB/c mice. The therapeutic intervention started after 3 days with the establishment of the tumor, in concentrations of 5 and 10 mg/kg (10 mg/kg was the maximum concentration considering the limitations of the solubility of the usnic acid) diluted in DMSO, in a total volume of 200 μL of PBS was administered via intraperitoneal injection (6 or 10 times in 2 weeks), and each mouse was analyzed by optical image on days 2, 9 and 16 after inoculation. After the final analysis of the images, the liver tissues and disseminated peritoneal tumors were excised and counted from the control and treatments. The groups showed different numbers of tumor nodules of varying sizes and in the quantitative analysis of the metastasis score the authors did not observe a statistically significant difference in the metastasis score between the control and treatment groups; in addition, the authors describe tumor progression as evidenced by increased bioluminescence. Thus, they concluded that usnic acid had no significant inhibitory effect on metastasis in the orthotopic murine colorectal cancer model, although they observed inhibitory activity of usnic acid against cell invasion in vitro experiments for CT26 cells (Table 1).

Regarding in vitro cytotoxic activity on the same cancerous cell lines from humans and mice and cell invasion, treated with 5 μM potassium usnate, Yang et al. [59] observed that potassium usnate showed cytotoxic activity and inhibited invasion in colorectal cancer cells. At the level of comparison of both molecules, the authors calculated the IC_50_ and observed a significantly lower value for potassium usnate than for the usnic acid molecule, except in SW480 and CT26 cells where there was no significant difference. Potassium usnate also showed a more potent inhibitory activity against cell invasion than the usnic acid in Caco2 and HCT116 cells. The authors further suggest that potassium usnate retains cytotoxicity and invasive inhibitory activity of usnic acid (Table 1).

In in vivo experimentation seeking to evaluate the inhibitory activity of potassium usnate against metastasis in vivo Yang et al. [59] performed the same experimental protocol used for usnic acid, with the exception that the dose of 20 mg/kg was added and the solubilization was performed in distilled water. The authors observed a significantly smaller number of tumor nodules for the treated groups, in the quantitative analysis and in the histological examination, they showed that the metastasis score and the tumor area were significantly lower in the group treated with 20 mg/kg. In addition, the authors still describe the immunohistochemical analysis that the nuclear staining of the mitosis marker, phosphorylated histone H3 (pHH3), was lower in the group treated with 20 mg/kg than in the other groups. In the optical image analysis, the region of interest showed a much weaker bioluminescent signal in the group treated with 20 mg/kg of potassium usnate than in the other groups. Thus, the authors suggest that potassium usnate inhibited tumor growth in a murine model with orthotopic liver metastasis.

Regarding the bioavailability for usnic acid and potassium usnate, Yang et al. [59] evaluated the liver tissues, isolated tumor and blood plasma using the LC-MS/MS analysis. They noted that the amount and speed of absorption of potassium usnate were significantly greater than that of usnic acid. These results led the authors to suggest that water solubility was a limiting factor of oral bioavailability making it available for its performance at the target site of action and that potassium usnate showed high potential as a drug candidate (Table 2).

**Table 1:** Biological applications of usnic acid and potassium usnate in vitro.

Molluscicidal Activity on *Biomphalaria glabrata*				Reference
**Usnic Acid (μg/mL)**				
**Embryonic Stages**	**LC_50_**	**LC_90_**	**LC_100_**	
Blastula	1.38	1.62	2.0	[26,27]
Gastrula	3.47	4.45	4.5	
Trocophore	5.11	5.36	6.0	
Veliger	2.93	4.49	6.0	
Adult	2.12	3.45	4.0	
**Potassium Usnate (μg/mL)**				
Blastula	4.97	5.41	6.0	[35,36]
Gastrula	3.11	3.58	4.0	
Trocophore	3.55	4.44	4.5	
Veliger	2.67	3.89	4.5	
Adult	0.92	-	1.0	[28]
**Schistosomicidal Activity on *Schistosoma mansoni***				
	**Usnic Acid (μg/mL)**			
	**LC_50_**	**LC_90_**	**LC_100_**	
Cercariae	NI	NI	10 (90 min)	[47]
Adult	NI	NI	200 (120 h)	[50]
	**Potassium Usnate (μg/mL)**			
Cercariae	1.98 (60 min)	4.93 (60 min)	5 (120 min)	[35,36]
Adult	50 (24 h)	12.5 (96 h)	100 (24 h)	[37,39]
**Antileishmania Activity**				
**Phase**	**Usnic Acid (μM)**	**Potassium Usnate (μM)**		
Promastigote (IC_50_)	18.30	2.99		[52]
**Antitumor Activity**				
**Mean Cytotoxicity of Cancer Cells**				
**Lineage**	**Molecule Analyzed**			
	**Usnic Acid (µM)**	**Potassium Usnate (µM)**		[59]
HCT116	97.4	87.0		
DLD1	96.0	67.5		
SW480	84.0	94.0		
HT29	68.5	57.4		
SW620	46.3	32.0		
Caco2	38.5	25.0		
CT26	38.4	35.0		
COL320	94.0	59.6		
**Mean Invasive Ability of Cancer Cells after Drug Treatment**				
	**Usnic Acid (µM)**	**Potassium Usnate (µM)**		
CaCo2	73.0	65.0		
HCT116	64.0	52.0		
CT26	80.5	79.5		

NI: value/concentration not informed. LC: lethal concentration. IC: inhibitory concentration.

## 6. Behavioral Changes and Toxicity

The acute toxicity test is performed to safely determine the dosage ranges of substances, but it can also provide initial information about the mechanisms of behavioral changes or even toxicity [60]. Acute oral toxicity for usnic acid is reported only by the company Sigma-Aldrich^®^ [61], although it does not provide methodological details of the behavioral and toxicological effects caused by usnic acid in vivo, Sigma-Aldrich^®^ states that the LD_50_ for usnic acid corresponds at 838 mg/kg in a murine model. However, Abo-Khatwa et al. [62] evaluated the acute toxicity of usnic acid at a dose of 80–280 mg/kg administered subcutaneously in mice. The LD_50_ of usnic acid was 180 mg/kg and behavioral changes included long achalasia, ponopalmosis, or spastic paralysis. These symptoms were most evident 2–5 h after treatment.

Joseph et al. [63] conducted experimental research whose objective was to evaluate the mechanisms of action of usnic acid and its effects on mitochondria; the parameters analyzed were measurement of the rate of oxygen consumption and/or generation of (ATP) adenosine triphosphate associated with the mitochondrial structure and liver functions of female B6C3F1 rats. The isolated diet started at the 8th week of age; the rats received usnic acid at concentrations of 0, 60, 180, and 600 µg/mL, for 14 days. The analyses showed a significant effect of usnic acid on the expression of several genes only at the highest concentration. The results showed significant induction of genes associated with complexes I–IV of the electron transport chain, and other genes involved in the oxidation of fatty acids, in the Krebs cycle, cell apoptosis, and in membrane transporters are also expressed. The toxicity of usnic acid has also been reported for other small and medium-sized animals; the LD_50_ intravenously corresponded to 25 mg/kg in mice, 30 mg/kg in rats and rabbits, 40 mg/kg in dogs, and 485–647 mg/kg in domestic sheep [2,64].

The acute oral toxicity of potassium usnate was reported by Araújo et al. [38] using Swiss Webster mice and the experiment was carried out according to the guidelines of the Organization for Economic Cooperation and Development (OECD). The mice were divided into 4 groups: a control group that received autoclaved water and three groups that were treated with potassium usnate at doses of 500, 1000 and 2000 mg/kg. The mice were examined for 5 days; on the first day, behavioral changes were observed every 10 min for 4 h, followed by two observations each day seeking to record behavioral and toxicity parameters. The authors did not observe any behavioral changes in the negative control group, while all concentrations tested showed at least some effect, be it stimulating, depressive, or changes in depression versus agitation, with emphasis on the concentration of 2000 mg/kg that presented the greatest number of changes, such as increased respiratory rate, piloerection, stereotyped movement, fine tremors, the elevation of the upper train, spasms, prostration, reduction of the hindquarters, photophobia, fecal excretions and abdominal distention. On the other hand, they observed a reversal of these initial clinical signs after 1 h of administration.

Regarding the effects of acute toxicity of potassium usnate Araújo et al. [38] observed deaths of only mice treated at a dose of 2000 mg/kg at intervals of 48 and 72 h, corresponding to 40%. Finally, although the authors were unable to calculate the LD_50_ of the potassium usnate, it showed considerably less toxicity when compared to the usnic acid. (Table 2).

## 7. Conclusions and Perspectives

The analogy of scientific research between usnic acid and its derivative of potassium usnate provides us with relevant information on upgrading the biological, pharmacological, and toxic activities of the usnic acid derivative. The set of this information is promising and enables new strategies and/or interests in the development of biotechnological resources for applications of potassium usnate in several other biological models or even generating patents.

## Figures and Tables

**Table 2:** Biological applications of usnic acid and potassium usnate in vivo.

Antinociceptive Activity			References
**Usnic Acid**			
**Concentration (mg/kg)**	**Effect (%)**		
30	-		[55]
100	60		
**Potassium Usnate**			
**Concentration (mg/kg)**	**Effect (%)**		
10	69		[38]
20	78		
**Antitumor Activity and Bioavailability**			
**Mean of Distribution, in the Tumor, Liver and Blood Plasma**			[59]
**Tissue**	**Molecule Analyzed**		
	**Usnic Acid**	**Potassium Usnate**	
Tumor nmole/g	0.0	1.5	
Liver nmole/g	0.28	2.6	
Plasma µM	0.2	1.7	
**Mean Levels of Gene Expression of EMT Markers in Caco2 Cells Treated with the Drugs**			
	**Usnic Acid**	**Potassium Usnate**	
E-cad	0.98	0.78	
N-cad	0.84	0.74	
Snail	0.49	0.53	
Twist	0.63	0.64	
Slug	0.75	0.62	
ZEB1	0.95	0.74	
ZEB2	0.82	0.73	
**Mean Levels of mRNA Expression of Genes Related to Cell Motility in Caco2 Cells Treated with the Drugs**			
	**Usnic Acid**	**Potassium Usnate**	
CANP1	0.95	0.74	
CDC42	0.90	0.55	
CFL1	0.78	0.70	
IGF1	0.77	0.70	
WASF1	1.03	0.71	
WASL	1.00	0.53	
**Acute Toxicity**			
**Usnic Acid**			
**Animals**	**Concentration (mg/kg)**	**LD_50_(mg/kg)**	
Mice	NI	25	[2]
Rats and rabbits	NI	30	
Dogs	NI	40	
Sheep	NI	485 e 647	[64]
Mice	80–280	180	[62]
Mice	NI	838	[61]
Potassium Usnate			
Mice	500, 1000 and 2000	> 2000	[38]

NI: value/concentration not informed. LD_50_: lethal dose 50%.

## References

[1] Ahti T., Stenroos S., Xavier Filho L. (1993). The lichen family Cladoniaceae in Paraíba, Pernambuco and Sergipe, Northeast Brazil. Trop. Biol..

[2] Ingólfsdóttir K. (2002). Usnic acid. Phytochemistry.

[3] Calcott M.J., Ackerley D.F., Knight A., Keyzers R.A., Owen J.G. (2018). Secondary metabolism in the lichen symbiosis. Chem. Soc. Rev..

[4] Galanty A., Paśko P., Podolak I. (2019). Enantioselective activity of usnic acid: A comprehensive review and future perspectives. Phytochem. Rev..

[5] Ranković B., Kosanić M. (2021). Biotechnological substances in lichens. Natural Bioactive Compounds..

[6] Cocchietto M., Skert N., Nimis P.L., Sava G. (2002). A review on usnic acid, an interesting natural compound. Naturwissenschaften.

[7] Cazarin C.A., Dalmagro A.P., Gonçalves A.E., Boeing T., Silva L.M., Corrêa R., Klein-Júnior L.C., Pinto B.C., Lorenzett T.S., Sobrinho T.U.C. (2021). Usnic acid enantiomers restore cognitive deficits and neurochemical alterations induced by Aβ_1-42_ in mice. Behav. Brain Res..

[8] Huneck S., Yoshimura I. (1996). Identification of Lichen Substances.

[9] Macedo D.C.S., Almeida F.J.F., Wanderley M.S.O., Ferraz M.S., Santos N.P.S., López A.M.Q., Santos-Magalhães N.S., Lira-Nogueira M.C.B. (2020). Usnic acid: From an ancient lichen derivative to promising biological and nanotechnology applications. Phytochem. Rev..

[10] Bustinza F. (1952). Antibacterial substances from lichens. Econ. Bot..

[11] Rafanelli S., Bacchilega R., Stanganelli I., Rafanelli A. (1995). Contact dermatitis from usnic acid in vaginal ovules. Contact Dermatitis..

[12] Rancan F., Rosan S., Boehm K., Fernández E., Hidalgo M.E., Quihot W., Rubio C., Boehm F., Piazena H., Oltmanns U. (2002). Protection against UVB irradiation by natural filters extracted from lichens. J. Photochem. Photobiol. B..

[13] Nybakken L., Julkunen-Tiitto R. (2006). UV-B induces usnic acid in reindeer lichens. Lichenologist.

[14] Goel M., Kalra R., Ponnan P., Jayaweera J.A.A.S., Kumbukgolla W.W. (2021). Inhibition of penicillin-binding protein 2a (PBP2a) in methicillin resistant *Staphylococcus aureus* (MRSA) by combination of oxacillin and a bioactive compound from *Ramalina. roesleri*. Microb. Pathog..

[15] Kumar K., Mishra J.P.N., Singh R.P. (2020). Usnic acid induces apoptosis in human gastric cancer cells through ROS generation and DNA damage and causes up-regulation of DNA-PKcs and γ-H2A.X phosphorylation. Chem. Biol. Interact..

[16] Chelombitko M.A., Firsov A.M., Kotova E.A., Rokitskaya T.I., Khailova L.S., Popova L.B., Chernyak B.V., Antonenko Y.N. (2020). Usnic acid as calcium ionophore and mast cells stimulator. Biochim. Biophys. Acta Biomembr..

[17] Sokolov D.N., Zarubaev V.V., Shtro A.A., Polovinka M.P., Luzina O.A., Komarova N.I., Salakhutdinov N.F., Kiselev O.I. (2012). Anti-viral activity of (-)/(+) usnic acids and their derivatives against influenza virus A(H1N1) 2009. Bioorg. Med. Chem. Lett..

[18] Kumar P., Ramtekeb P.W., Pandeyc A.C., Pandey H. (2019). Evaluation of antifungal activity of blended cinnamon oil and usnic acid nanoemulsion using candidiasis and dermatophytosis models. Biocatal. Agric. Biotechnol..

[19] Lee S., Lee Y., Ha S., Chung H.Y., Kim H., Hur J.S., Lee J. (2020). Anti-inflammatory effects of usnic acid in an MPTP-induced mouse model of Parkinson’s disease. Brain Res..

[20] Si K., Wei L., Yu X., Wu F., Li X., Li C., Cheng Y. (2016). Data on (+)-usnic acid: A new application to treat toxoplasmosis. Data Brief..

[21] Zakharenko A.L., Luzina O.A., Sokolov D.N., Kaledin V.I., Nikolin V.P., Popova N.A., Patel J., Zakharova O.D., Chepanova A.A., Zafar A. (2019). Novel tyrosyl-DNA phosphodiesterase 1 inhibitors enhance the therapeutic impact of topotecan on in vivo tumor models. Eur. J. Med. Chem..

[22] Kristmundsdóttir T., Jónsdóttir E., Ogmundsdóttir H.M., Ingólfsdóttir K. (2005). Solubilization of poorly soluble lichen metabolites for biological testing on cell lines. Eur. J. Pharm. Sci..

[23] Jin J., Rao Y., Bian X., Zeng A., Yang G. (2013). Solubility of (+)-usnic acid in water, ethanol, acetone, ethyl acetate and n-hexane. J. Solution Chem..

[24] Chen S., Liu H., Ye W., Li S., Li D., Liu Z., Zhang W. (2020). Ochuscins A-G, highly oxygenated usnic acid derivatives from the deep-sea-derived fungus *Ochroconis* sp. FS449. Tetrahedron..

[25] Shi C.J., Peng W., Zhao J.H., Yang H.L., Qu L.L., Wang C., Kong L.Y., Wang X.B. (2020). Usnic acid derivatives as tau-aggregation and neuroinflammation inhibitors. Eur. J. Med. Chem..

[26] Araújo H.D.A., Silva L.R.S., Siqueira W.N., Fonseca C.S.M., Silva N.H., Melo A.M.M.A., Martins M.C.B., Lima V.L.M. (2018). Toxicity of usnic acid from *Cladonia substellata* (Lichen) to embryos and adults of *Biomphalaria glabrata*. Acta Trop..

[27] Araújo H.D.A., Silva L.R.S., Siqueira W.N., Fonseca C.S.M., Silva N.H., Melo A.M.M.A., Martins M.C.B., Lima V.L.M. (2018). Dataset on usnic acid from *Cladonia substellata* Vainio (Lichen) schistosomiasis mansoni’s vector control and environmental toxicity. Data Brief..

[28] Martins M.C.B., Silva R.L., Barbosa P.S., Rodrigues B.R.M., Albuquerque A.C., Falcão P.S., Lima V.L.M., Silva N.H., Pereira E.C. (2018). Effects of usnic, barbatic and fumarprotocetraric acids on survival of *Nasutitermes corniger* (Isoptera: Termitidae: Nasutitermitinae). Sociobiology..

[29] Martins M.C.B., Lima M.J.G., Santiago R., Buril M.L.L., Pereira E.C., Legaz M.E., Vicente C., Silva N.H. (2017). New biotechnological methods for producing therapeutic compounds (Usnic, Stictic and Norstictic acids) by cell immobilization of the lichen *Cladonia substellata* Vainio. Biotechnol. Ind. J..

[30] Santos F.T.J., Siqueira W.N., Santos M.L.O., Silva H.A.M.F., Sá J.L.F., Fernandes T.S., Silva N.H., França E.J., Silva E.B., Melo A.M.M.A. (2018). Radiosensitizer effect of usnic acid on *Biomphalaria glabrata* embryos. Int. J. Radiat. Biol..

[31] Santiago R., Martins M.C.B., Vilaça M.D., Barros L.F.B., Nascimento T., Silva N.H., Falcão E.P.S., Legaz M.E., Vicente C., Pereira E.C. (2018). Phytochemical and biological evaluation of metabolites produced by alginate-immobilized bionts isolated from the lichen *Cladonia substellata* vain. Fitoterapia.

[32] Silva C.R., Marinho K.S.N., Silva T.D.S., Ferreira D.K.S., Aguiar G.M., Martins M.C.B., Santos K.R.P., Aguiar Júnior F.C.A., Santos N.P.S., Pereira E.C. (2017). Teratogenic effect of usnic acid from *Cladonia substellata* Vainio during organogenesis. BioMed Res. Int..

[33] Luz J.S.B., Oliveira E.B., Martins M.C.B., Silva N.H., Alves L.C., Santos F.A.B., Silva L.L.S., Silva E.C., Medeiros P.L. (2015). Ultrastructural analysis of *Leishmania infantum chagasi* promastigotes forms treated in vitro with usnic acid. Sci. World J..

[34] Martins M.C.B., Silva M.C., Silva L.R.S., Lima V.L.M., Pereira E.C., Falcão E.P., Melo A.M.M.A., Silva N.H. (2014). Usnic acid potassium salt: An alternative for the control of *Biomphalaria glabrata* (Say, 1818). PLOS ONE.

[35] Araújo H.D.A., Melo A.M.M.A., Siqueira W.N., Martins M.C.B., Aires A.L., Albuquerque M.C.P.A., Silva N.H., Lima V.L.M. (2018). Potassium usnate toxicity against embryonic stages of the snail *Biomphalaria glabrata* and *Schistosoma mansoni* cercariae. Acta Trop..

[36] Araújo H.D.A., Melo A.M.M.A., Siqueira W.N., Martins M.C.B., Aires A.L., Albuquerque M.C.P.A., Silva N.H., Lima V.L.M. (2018). Dataset on schistosomiasis control using potassium usnate against *Biomphalaria glabrata* at different developmental stage and *Schistosoma mansoni* cercariae. Data Brief..

[37] Araújo H.D.A., Aires A.L., Soares C.L.R., Brito T.G.S., Nascimento W.M., Martins M.C.B., Silva T.G., Brayner F.A., Alves L.C., Silva N.H. (2019). Usnic acid potassium salt from *Cladonia substellata* (Lichen): Synthesis, cytotoxicity and in vitro anthelmintic activity and ultrastructural analysis against adult worms of *Schistosoma mansoni*. Acta Trop..

[38] Araújo H.D.A., Silva Júnior J.G., Oliveira J.R.S., Ribeiro M.H.M.L., Martins M.C.B., Bezerra M.A.C., Aires A.L., Albuquerque M.C.P.A., Melo-Júnior M.R., Pontes Filho N.T. (2019). Usnic acid potassium salt: Evaluation of the acute toxicity and antinociceptive effect in murine model. Molecules.

[39] Araújo H.D.A., Silva N.H., Albuquerque M.C.P.A., Aires A.L., Lima V.L.M. (2020). Potassium usnate, a water-soluble usnic acid salt, shows enhanced activity against *Schistosoma mansoni* in vitro. Exp. Parasitol..

[40] Araújo H.D.A., Santos V.H.B., Brayner F.A., Alves L.C., Silva N.H., Albuquerque M.C.P.A., Aires A.L., Lima V.L.M. (2020). In vitro activity of usnic acid potassium salt against different developmental stages of *Schistosoma mansoni*: An ultrastructural study. Acta Trop..

[41] Buemi G., Zuccarello F. (1990). Molecular conformations, hydrogen-bond strengths and electronic structure of usnic acid: An AM1 and CNDO/S study. J. Mol. Struc.-Theochem..

[42] Galasso V. (2010). Probing the molecular and electronic structure of the lichen metabolite usnic acid: A DFT study. Chem. Phys..

[43] Guo L., Shi Q., Fang J.L., Mei N., Ali A.A., Lewis S.M., Leakey J.E., Frankos V.H. (2008). Review of usnic acid and Usnea barbata toxicity. J. Environ. Sci. Health. C Environ. Carcinog. Ecotoxicol. Rev..

[44] Scholte R.G., Gosoniu L., Malone J.B., Chammartin F., Utzinger J., Vounatsou P. (2014). Predictive risk mapping of schistosomiasis in Brazil using Bayesian geostatistical models. Acta Trop..

[45] Araújo H.D.A., Silva H.A.M.F., Siqueira W.N., Santos V.H.B., Lima M.V., Silva Júnior J., Silva N.H., Albuquerque M.C.P.A., Melo A.M.M.A., Aires A.L. (2021). Sublethal concentrations of usnic acid potassium salt impairs physiological parameters of *Biomphalaria glabrata* (Say, 1818) (Pulmonata: Planorbidae) infected and not infected with *Schistosoma mansoni*. Acta Trop..

[46] Famakinde D.O. (2017). Molecular context of *Schistosoma mansoni* transmission in the molluscan environments: A mini-review. Acta Trop..

[47] Carvalho A.N., Melo A.M.M.A., Amâncio F.F., Araújo H.D.A., Silva H.A.M.F., Albuquerque M.C.P.A., Aires A.L., Martins M.C.B., Silva N.H. (2015). Avaliação da atividade do ácido úsnico sobre cercárias de Schistosoma mansoni. XXIV Congresso da Sociedade Brasileira de Parasitologia. XXIII Congresso Latinoamericano de Parasitologia..

[48] Katz N. (2008). The discovery of schistosomiasis mansoni in Brazil. Acta Trop..

[49] World Health Organization (2017). Weekly Epidemiological Record. Schistosomiasis and Soil-transmitted Helminthiases: Number of People Treated in 2016. http://apps.-who.int/iris/bitstream/handle/10665/259593/WER9249.pdf?sequence=1.

[50] Salloum A.I.O., Lucarini V.R., Tozatti M.G., Medeiros J., Silva M.L.A., Magalhães L.G., Cunha W.R. (2012). In vitro schistosomicidal activity of *Usnea steineri* extract and its major constituent (+)-usnic acid against *Schistosoma mansoni*. Planta Med..

[51] Charyyeva A., Çetinkaya Ü., Özkan B., Şahin S., Yaprak N., Şahin I., Yurchenko V., Kostygov A.Y. (2021). Genetic diversity of *Leishmania tropica*: Unexpectedly complex distribution pattern. Acta Trop..

[52] Luz J.S.B. (2019). Análise Comparativa Da Atividade Leishmanicida In Vitro Do Ácido Úsnico E Do Seu Derivado Usnato De Potássio Isolado Da *Cladonia Substellata* Vainio. Ph.D. Thesis.

[53] DeSantana J.M., Perissinotti D.M.N., Oliveira Junior J.O., Correia L.M.F., Oliveira C.M., Fonseca P.R.B. (2020). Revised definition of pain after four decades. BrJP..

[54] Raja S.N., Carr D.B., Cohen M., Finnerup N.B., Flor H., Gibson S., Keefe F.J., Mogil J.S., Ringkamp M., Sluka K.A. (2020). The revised international association for the study of pain definition of pain: Concepts, challenges, and compromises. Pain..

[55] Okuyama E., Umeyama K., Yamazaki M., Kinoshita Y., Yamamoto Y. (1995). Usnic acid and diffractaic acid as analgesic and antipyretic components of *Usnea diffracta*. Planta Med..

[56] Prokopiev I.A., Filippov E.V., Filippova G.V., Gladkina N.P. (2017). Genotoxic effect of usnic acid enantiomers in vitro in human peripheral blood lymphocytes. Tsitologiia..

[57] O’Connell E., Reynolds I.S., McNamara D.A., Burke J.P., Prehn J.H.M. (2021). Resistance to cell death in mucinous colorectal cancer - A review. Cancers.

[58] Rubio J., Cristóbal I., Santos A., Caramés C., Luque M., Sanz-Alvarez M., Zazo S., Madoz-Gúrpide J., Rojo F., García-Foncillas J. (2021). Low microRNA-19b expression shows a promising clinical impact in locally advanced rectal cancer. Cancers.

[59] Yang Y., Bae W.K., Lee J.Y., Choi Y.J., Lee K.H., Park M.S., Yu Y.H., Park S.Y., Zhou R., Taş İ. (2018). Potassium usnate, a water-soluble usnic acid salt, shows enhanced bioavailability and inhibits invasion and metastasis in colorectal cancer. Sci. Rep..

[60] Veras B.O., Oliveira J.R.S., Lima V.L.M., Navarro D.M.A.F., Aguiar J.C.R.O.F., Moura G.M.M., Silva J.W., Assis C.R.D., Gorlach-Lira K., Assis P.A.C. (2021). The essential oil of the leaves of *Verbesina macrophylla* (Cass.) S.F.Blake has antimicrobial, anti-inflammatory and antipyretic activities and is toxicologically safe. J. Ethnopharmacol..

[61] Sigma-Aldrich, Safety Data Sheet. https://www.sigmaaldrich.com/MSDS/MSDS/DisplayMSDSPage.do?country=BR&language=pt&productNumber=329967&brand=ALDRICH&PageToGoToURL=https%3A%2F%2Fww.sigmaaldrich.com%2Fcatalog%2Fsearch%3Fterm%3D7562610%26interface%3DCAS%2520No.%26N%3D0%26mode%3Dpartialmax%26lang%3Dpt%26region%3DBR%26focus%3Dproduct.

[62] Abo-Khatwa A.N., Al-Robai A.A., Al-Jawhari D.A. (2015). The uncoupling of oxidative phosphorylation of mouse-liver mitochondria in vivo by usnic acid. JKAU..

[63] Joseph A., Lee T., Moland C.L., Branham W.S., Fuscoe J.C., Leakey J.E.A., Allaben W.T., Lewis S.M., Ali A.A., Desai V.G. (2009). Effect of (+)-usnic acid on mitochondrial functions as measured by mitochondria-specific oligonucleotide microarray in liver of B6C3F1 mice. Mitochondrion.

[64] Dailey R.N., Montgomery D.L., Ingram J.T., Siemion R., Vasquez M., Raisbeck M.F. (2008). Toxicity of the lichen secondary metabolite (+)-usnic acid in domestic sheep. Vet. Pathol..

